# Pulmonary arterial hypertension post COVID-19: A sequala of SARS-CoV-2 infection?

**DOI:** 10.1016/j.rmcr.2021.101429

**Published:** 2021-05-12

**Authors:** Abdul Wali Khan, Irfan Ullah, Kiran Shafiq Khan, Muhammad Junaid Tahir, Sri Masyeni, Harapan Harapan

**Affiliations:** a Department of Medicine, Hayatabad Medical Complex, Peshawar, 25000, Pakistan; b Kabir Medical College, Gandhara University, Peshawar, 25000, Pakistan; c Dow Medical College, Dow University of Heath Sciences, Karachi, 74200, Pakistan; d Ameer-ud-Din Medical College, Affiliated with University of Health and Sciences, Lahore, 54000, Pakistan; e Lahore General Hospital, Lahore, 54000, Pakistan; f Department of Internal Medicine, Faculty of Medicine and Health Sciences Universitas Warmadewa, Denpasar, Bali, 80235, Indonesia; g Department of Internal Medicine, Sanjiwani Hospital, Denpasar, Bali, 80235, Indonesia; h Medical Research Unit, School of Medicine, Universitas Syiah Kuala, Banda Aceh, 23111, Indonesia; i Tropical Diseases Centre, School of Medicine, Universitas Syiah Kuala, Banda Aceh, 23111, Indonesia; j Department of Microbiology, School of Medicine, Universitas Syiah Kuala, Banda Aceh, 23111, Indonesia

**Keywords:** COVID-19, Pulmonary arterial hypertension, PAH, Sequala, Complication

## Abstract

It has been suggested that pulmonary arterial hypertension (PAH) could be a potential sequela of coronavirus disease 2019 (COVID-19) in particular in those with hypertension; however, development of PAH after the course of COVID-19 in normotensive individuals are rarely reported. Here, we report a patient who developed PAH two months post-COVID-19. The patient was a 55-year-old female and normotensive, tested positive for severe acute respiratory syndrome coronavirus 2 (SARS-CoV-2), developed mild respiratory distress syndrome and necessitated continuous positive airway pressure during the treatment in the hospital. After two months discharged from the hospital with RT-PCR negative for SARS-CoV-2, the patient presented with exertional dyspnea, dry cough, fatigue and episodes of syncope during exertion. Based on clinical presentation, electrocardiography, computed tomography, and transthoracic echocardiography assessment, PAH diagnosis was made. To our knowledge, this is a rare PAH case and this highlights the possible of PAH as sequala that might present in post COVID-19 patients.

## 1 Introduction

The current coronavirus virus 2019 (COVID-19) pandemic, cause by severe acute respiratory syndrome coronavirus 2 (SARS-CoV-2), has infected over 123 million people worldwide with more than 2.7 million deaths as of March 22, 2021 based on COVID-19 Dashboard by Johns Hopkins University [1]. In most of SARS-CoV-2 infection, the symptoms remain mild with fever, cough and flu; however, it could develop to pneumonia and acute respiratory distress syndrome with several complications [[2], [3], [4], [5]]. COVID-19-associated cardiovascular complications such as myocardial injury, myocarditis, dysrhythmias, acute myocardial infarction, heart failure, venous thromboembolic events such as stroke, acute coronary syndrome and others have been reported [[6], [7], [8]].

Pulmonary arterial hypertension (PAH) is a chronic fatal disease that requires immediate and proper medical treatment since it could cause cardiopulmonary collapse [9,10]. Heart failure and chronic kidney disease are the most common underlying medical conditions of PAH [11]. It has been reported that COVID-19 patients are at risk of developing cardiac or pulmonary complications due to pulmonary hypertension [9]. To date, the pathophysiology and effects of COVID-19 in PAH are unclear. In this case report, we report the case of a severe COVID-19 patient, had no PAH underlying medical conditions, who later developed PAH after two months discharged from the hospital.

## 2 Case presentation

A 55-year-old female, with no previous PAH comorbidities, initially presented with fever, shortness of breath, dry cough, chest pain, nausea, and generalized body aches for seven days. On presentation in the hospital, the blood pressure was 100/70 mmHg, pulse was 105 bpm, respiratory rate of 22/min, and oxygen saturation was 77%. On examination, there were bilateral coarse crackles in both lower lung fields. Based on the history and examination findings, a COVID-19 was suspected. The patient was admitted to the COVID-19 isolation unit and was started on oxygen by a simple face mask (5 L). The patient was started on intravenous (IV) azithromycin (500 mg/day) and ceftriaxone (75 mg/kg/day IV) along with enoxaparin sodium (40 mg for each 12 hours subcutaneously), dexamethasone (6 mg/day IV), vitamin C and Zinc. The baseline lab testing and a chest X-ray (CXR) were ordered. Moreover, a nasopharyngeal swab was collected for SARS-CoV-2 RT-PCR.

Her initial lab reports showed hemoglobin 11 g/dL, WBCs 17000/mm^3^ with polymorphonuclear neutrophils 90% and lymphocyte 7%, LDH 900U/L, serum ferritin 1100 ng/mL, CRP 12 mg/L, PT 14 sec, INR 1.1, aPTT 32 sec, ALT 57 U/L, AST 78 U/L, serum bilirubin total 2 mg/dl, serum creatinine 1.1 mg/dL, and blood urea 40 mg/dL. Her CXR demonstrated bilateral infiltrates. RT-PCR result confirmed as SARS-CoV-2-positive. Arterial blood gas analysis was consistent with hypoxemia and respiratory alkalosis (pH 7.6, PaO_2_ 42 mmHg, and PaCO_2_ 22 mmHg). Over the course of treatment, the patient developed mild respiratory distress syndrome (PaO_2_/FiO_2_ 250) and necessitated continuous positive airway pressure (CPAP). The patient remained in the COVID-19 isolation unit for 18 days. On discharge, patient had improved clinically with oxygen saturation was 92% without oxygen and SARS-CoV-2 RT-PCR was negative.

**Fig. 1:**
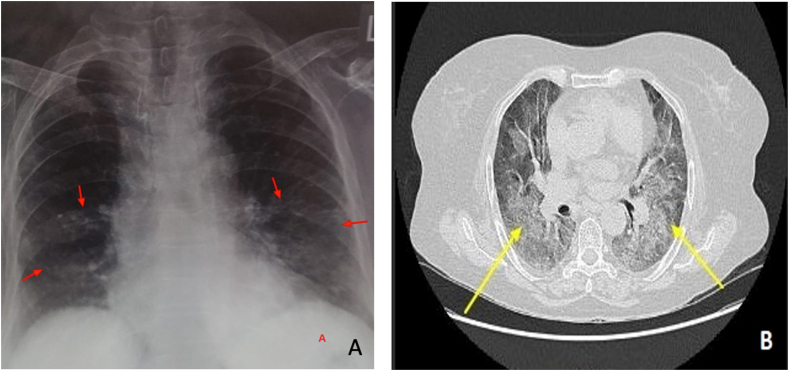
(A). X ray of a post-COVID-19 patient. Red arrows indicate fibrotic changes, more prominent in the basal regions of both lungs. (B). High resolution CT-scan suggest massive fibrosis, diffuse symmetrical ground glass opacities (yellow arrows) involving both lung fields along with septal thickening. (For interpretation of the references to colour in this figure legend, the reader is referred to the Web version of this article.)

Two months after discharge, she visited the outpatient department with complaints of exertional dyspnea, dry cough, and fatigue for one month. She also reported an episode of exertional syncope. Her vitals revealed blood pressure of 140/90 mmHg, pulse rate 90 bpm, respiratory rate of 18/min, and oxygen saturation of 88% without any oxygen support. On examination, her jugular venous pressure raised 13 cm of H_2_O; left parasternal heave, and wide splitting of the second heart sound was noted. All the relevant blood tests were ordered along with a CXR, electrocardiography (ECG), and a SARS-CoV-2 RT-PCR. The ECG suggested right axis deviation, and the blood test results are presented in Table 1. A RT-PCR for SARS-CoV-2 was negative which ruled out re-infection. The lung CXR demonstrated right atrial enlargement and some fibrotic changes (Fig. 1A). A high-resolution CT-scan revealed massive fibrosis, diffuse symmetrical ground glass opacities involving both lung fields along with septal thickening and traction bronchiectasis most consistent with post-COVID-19 changes (Fig. 1B).

**Table 1:** Laboratory investigations.

Investigation	Value	Investigation	Value
White blood cells	14000/mm^3^	Lactate dehydrogenase	410 U/L
Neutrophils	78%	Serum ferritin	450 ng/mL
Lymphocytes	15%	C reactive protein	4.5 mg/L
Serum creatinine	1.3 mg/dL	Erythrocyte sedimentation rate	25/first hour
Serum urea	56 mg/dL	Prothrombin time, INR	13 sec, 1.2
Alanine transaminase	56 U/L	APTT	28 sec
Aspartate transaminase	43 U/L	Pro-calcitonin	1 ng/mL
Alkaline phosphatase	110 U/L	Serum sodium	138 mg/dL
D-Dimer	210 ng/mL	Serum potassium	4.3 mg/dL
Interleukin-6	18 pg/mL	Serum chloride	98 mg/dL

INR: International normalized ratio.

APTT: Activated partial thromboplastin time.

To further investigate the cause of patient problem, transthoracic echocardiography was ordered which showed right ventricular systolic blood pressure (RVSP) 59 mmHg, tricuspid regurgitant jet velocity (TRV) 4.5 m/s, pulmonary artery diameter was 31 mm with normal left side heart parameters. Based on echocardiographic findings and clinical presentation of the patient, the diagnosis of PAH group 3 was made in accordance with the European Society of Cardiology (ESC) and European Respiratory Society (ERS). The patient was commenced on epoprostenol infusion (25 ng/kg/min, repeated every week), montelukast 10 mg/day, oxygen therapy, and spironolactone 100 mg/day. In addition, all routine vaccinations including pneumococcal and influenza vaccines offered to the patient, and a follow-up visit was scheduled with a multidisciplinary team consisting of a pulmonologist, pulmonary rehabilitation team, and a cardiologist. Partial improvement was recorded in the subsequent visit (improved exercise tolerance, oxygen saturation, and decreased pulmonary arterial pressure to 45 mmHg).

## 3 Discussion

In this report, we aimed to highlight the possible relation of COVID-19 and PAH. Two months after recovering from COVID-19, the patient developed PAH with exertional dyspnea, dry cough, and fatigue as well as an episode of exertional syncope. One of the possible explanations is COVID-19 could cause pulmonary vasoconstriction that increased the pulmonary vascular resistance (PVR) [12]. The patient responded well to the initial treatment on her subsequent follow-up visit. This patient should be evaluated further for severe hypoxemia, thrombotic complications i.e. venous thromboembolic events, pulmonary micro-thrombi, vascular thickening, right ventricular hypertrophy and failure as they would be the predominant findings in patient developing PAH with COVID-19 [13].

It has been suggested that having history of hypertension increases the chances of PAH in patients with COVID-19 infection [14]. Therefore, patients with hypertension are at greater risk of developing PAH. One the biggest concern that need to addressed is did the patient have no PAH underling conditions that were potentially masked by the COVID-19? The patient reported in the present study was unique since that patient had no known comorbidities including hypertension. Assessment at the presentation and monitoring during COVID-19 infection revealed that the blood pressure ranged within normal limits. On physical examinations at both first presentation and during monitoring in the hospital suggested there were no signs of lower extremity deep vein thrombosis (DVT) and no sign of heart problems. Therefore, echocardiography and assessment of biomarkers for heart diseases were not advised. However, the D-dimer level was in the normal range (Table 1). Similarly, there was no evidence of rheumatologic diseases or other chronic illnesses on history and physical examination. We acknowledge that those assessments are critical to ensure that PAH in this patient was genuinely associated with COVID-19. However, due to the nature of the case, some of the underlying conditions of PAH could not confirmed.

Besides the above-mentioned limitation, PAH in case was diagnosed using a transthoracic echocardiography. The golden standard test to diagnose PAH is using right heart catheterization where PAH is defined with mean pulmonary artery pressure ≥25 mmHg, pulmonary artery wedge pressure <15 mmHg and pulmonary vascular resistance >3 units. However, transthoracic echocardiography results had enough parameters to suggested that the patient likely had PAH. Although direct association of SARS-CoV-2 infection and PAH is unable to be elucidated in this patient, this case highlights and informs the possible another sequala of COVID-19.

## 4 Conclusion

To our knowledge, this is a rare case of PAH that has not been previously reported. It is uncertain that either PAH is a risk factor of severe SARS-CoV-2 infection or vice versa. Further study is warranted to improve our understanding on the relationship between PAH and COVID-19 and therefore to be able to predict and to prevent PAH in COVID-19 patients.

## Consent for publication

Written informed consent for publication was sought from the patient.

## Funding

This study received no external funding.

## Authors’ contributions

AWK conceived and designed the study. AWK, IU, KSF, MJT were responsible for data collection and acquisition of data. AWK, IU, KSF, MJT, HH analyzed and/or interpreted the data. IU, HH wrote the initial manuscript. IU, HH critically revised the manuscript. All authors have read the final manuscript.

## Declaration of competing interest

The authors declare no conflicts of interest.
